# Development of a complex intervention for people with chronic pain after knee replacement: the STAR care pathway

**DOI:** 10.1186/s13063-017-2391-8

**Published:** 2018-01-23

**Authors:** Vikki Wylde, Nicholas Howells, Wendy Bertram, Andrew J. Moore, Julie Bruce, Candy McCabe, Ashley W. Blom, Jane Dennis, Amanda Burston, Rachael Gooberman-Hill

**Affiliations:** 1 Musculoskeletal Research Unit, Translational Health Sciences, Bristol Medical School, University of Bristol, Learning and Research Building, Southmead Hospital, Bristol, BS10 5NB UK; 2 National Institute for Health Research Bristol Biomedical Research Centre, University of Bristol, Bristol, UK; 3 North Bristol NHS Trust, Brunel Building, Southmead Hospital, Bristol, BS10 5NB UK; 4 Warwick Clinical Trials Unit, Warwick Medical School, University of Warwick, Coventry, CV4 7AL UK; 5 Department of Nursing and Midwifery, Faculty of Health and Applied Sciences, University of the West of England, Frenchay Campus, Coldharbour Lane, Bristol, BS16 1QY UK

**Keywords:** Total knee replacement, Chronic pain, Complex intervention development

## Abstract

**Background:**

Approximately 20% of people who have total knee replacement experience chronic pain afterwards, but there is little evidence about effective interventions for managing this type of pain. This article describes the systematic development and refinement of a complex intervention for people with chronic pain after knee replacement. The intervention is a care pathway involving an assessment clinic and onward referral, with telephone follow-up as required. In the design of this multistage study, we chose to focus on ensuring that the intervention was deliverable, implementable and acceptable.

**Methods:**

In line with the UK Medical Research Council’s recommendations for comprehensive development of complex interventions, multiple phases of work were undertaken. Following on from initial development work to design the intervention, the draft intervention content was refined through consensus questionnaires with 22 health professionals and discussion at meetings with 18 healthcare professionals. Testing of intervention delivery and acceptability to patients was undertaken by two health professionals delivering the assessment clinic to ten patients. Views about future implementation within the context of a randomised trial were evaluated through a questionnaire based on the Normalisation Measure Development (NoMAD) instrument with ten health professional stakeholders.

**Results:**

Consensus work with health professionals ensured the components of the intervention were appropriate and informed a number of substantive changes to improve the intervention. Testing of intervention delivery identified a number of logistical issues that were then addressed in the development of a comprehensive intervention training manual. Engagement with stakeholders indicated that the intervention could be successfully implemented in a clinical setting for evaluation in a randomised trial.

**Conclusions:**

This work has informed the development and refinement of a complex intervention for people with chronic pain after knee replacement. The next stage is to evaluate the clinical and cost-effectiveness of the STAR care pathway in a multicentre randomised trial.

**Electronic supplementary material:**

The online version of this article (10.1186/s13063-017-2391-8) contains supplementary material, which is available to authorized users.

## Background

Total knee replacement is a common elective surgical procedure, with over 70,000 primary total knee replacement operations performed annually in the UK National Health Service (NHS) [1]. People choose to undergo knee replacement in the hope that surgery will improve their pain [2]. Although primary total knee replacement is a successful intervention for reducing pain severity for most patients, about 20% of people experience chronic pain afterwards [3]. Chronic post-surgical pain is defined as pain that occurs or increases in intensity at 3 months or longer after surgery [4]. Chronic pain after total knee replacement is associated with functional limitations, decreased satisfaction and reduced quality of life [5].

The scale and impact [6] of chronic pain after knee replacement highlights the need for provision of adequate and high-quality healthcare. Unfortunately, current NHS service provision for people with chronic pain after knee replacement is patchy and inconsistent [7]. This reflects an absence of evidence about effective interventions for chronic post-surgical pain that could inform commissioning of services [8, 9] and emphasises the need to develop and evaluate interventions to address chronic pain after knee replacement. Chronic pain is challenging to treat [10] and there is a growing acknowledgement that a multidisciplinary and comprehensive approach is needed to provide appropriate and adequate management of chronic pain after knee replacement [11, 12]. It has been suggested that instead of developing novel interventions for chronic pain, there is a need to improve access to existing treatments and/or to deliver combination treatments that are matched to individual patients’ pain characteristics [10]. Currently there is a lack of clear pathways and referral processes for patients with chronic pain after knee replacement [7]. These are considered to be integral aspects of good care for chronic pain [10] and their absence hinders patients’ access to targeted and individualised care [13] indicating a pressing need for the development of new models of care delivery for this population.

This study aimed to address this through the development and refinement of a complex intervention for future testing within the framework of a pragmatic randomised controlled trial (the Support and Treatment After joint Replacement (STAR) trial). This intervention involves a novel assessment clinic and onward referral pathway for patients reporting moderate-severe pain at 3 months after total knee replacement, with telephone follow-up as required. The intervention aims to enable appropriate onwards referral to existing services to ensure that underlying reasons for chronic pain are considered and that treatment is targeted at these to improve pain management and to reduce the impact of pain. The initial design of this intervention was informed by a systematic review which evaluated the existing evidence for the management of chronic pain after total knee replacement [8], a survey of NHS service provision [7], qualitative work with health professionals [13], consensus meetings with pain experts, and patient and public involvement activities with a musculoskeletal forum [14]. Taken together, this background research informed the initial design of the STAR trial intervention.

This article describes the multiple phases and processes for the STAR trial intervention development and refinement, in keeping with the Medical Research Council’s (MRC) recommendations for comprehensive and transparent reporting on the development of complex interventions [15]. The study aimed to test that the intervention would be deliverable, implementable and acceptable. Specific objectives for this work were: (1) refinement of the draft intervention content, (2) testing of intervention delivery and acceptability to patients, and (3) evaluation of views about implementation of the intervention at the future trial centres. We report the final STAR trial intervention following guidance from the template for intervention description and replication (TIDieR) [16].

## Methods and Results

**Fig. 1:**
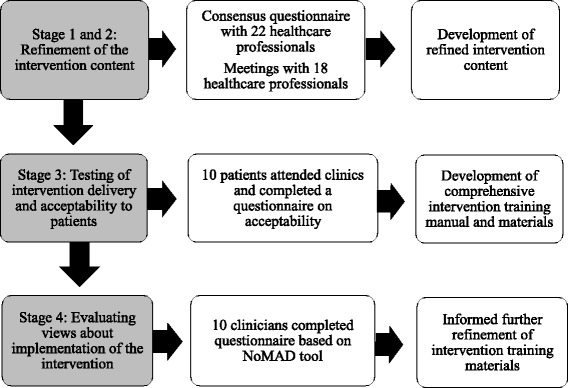
Overview of the intervention development process

Four stages of work were undertaken over a 12-month period. An overview of the intervention development process is provided in Fig. 1. Stages 1 and 2 involved consensus questionnaires and meetings with healthcare professionals to refine the intervention content. Stage 3 involved delivering the assessment clinics to patients to test intervention delivery and acceptability to patients. Stage 4 evaluated views about implementation of the STAR intervention with health professional stakeholders. Each of these stages is reported in more detail below.

Ethics approval was provided by the University of Bristol Faculty of Medicine and Dentistry Research Ethics Committee (stages 1 and 2; reference 21622) and NHS Research Ethics Committee (stage 3; reference 15/LO/2140). Relevant local Research and Development approvals were obtained and all participants provided informed, written consent. Stage 4 was conducted as stakeholder work with approval from the local NHS trust that this was appropriate.

### Stage 1: refinement of the STAR intervention content: consensus questionnaire with health professionals

Consensus work was conducted with healthcare professionals who had experience of providing care for patients with chronic pain after knee replacement. Participants were identified through participation in a previous study [17] and through informal networks at four orthopaedic centres. Twenty-two UK-based healthcare professionals took part: orthopaedic surgeons (*n* = 11), physiotherapists (*n* = 7), pain specialists (*n* = 2), one rheumatologist and one pain nurse. The sample size was deemed sufficient to elicit diverse views and establish consensus among the participants [18]. A questionnaire was developed to elicit opinions about the appropriateness of the individual components of the proposed STAR care pathway. Participants were provided with details of the draft STAR care pathway (Additional file 1) and asked to rank each component of the intervention from 1 to 9 (not appropriate to very appropriate). Participants were also provided with space to explain their ratings and make suggestions for additional components.

Mean appropriateness scores for the individual components of the draft STAR care pathway ranged from 7 to 8.5 (Table 1). Taking a mean score of 7–9 as agreement in keeping with the RAND/UCLA appropriateness criteria [19], all components of the intervention were considered appropriate.

**Table 1:** Stage 1 – health professionals’ (*n* = 22) appropriateness ratings for components included within the trial intervention

Component	Mean rating^a^	Range
Assessment clinic		
Clinics should be held with patients who have pain at 3 months or more after knee replacement	7.0	4–9
Assessment should be tailored to each individual patient	8.5	6–9
Conducted by a nurse or extended scope practitioner	7.5	5–9
Treatment should be guided by standardised assessment of pain	7.6	6–9
Standardised assessment of pain includes assessment of pain severity and impact	8.0	5–9
Possible onward referral pathways		
Monitoring (by a nurse or self-monitoring) as treatment	7.0	4–9
Referral to surgeon if signs of infection, malalignment or instability	8.5	6–9
Referral to General Practitioner if signs of depression	7.7	4–9
Referral to pain specialist if severe or interfering pain with indications of neuropathic or complex regional pain syndrome	7.9	5–9

^a^Mean appropriateness ratings ranked from 1 to 9 (not appropriate to very appropriate)

Free-text comments suggested improvements to the intervention, including: earlier screening of patients to ensure timely entry into the STAR care pathway, increased clarity over the purpose of follow-up, the addition of referrals to physiotherapy and to a general practitioner (GP) for anxiety management, and the importance of rapid access to suitable medications for neuropathic pain. Participants also emphasised the importance of a comprehensive intervention training package to ensure that a trained health practitioner eligible to deliver the trial intervention would be confident in their provision of post-operative knee specific tests and examinations.

The refined STAR care pathway and a summary report were then sent to all participants who were invited to contact the study team if they had further suggestions about content. None were received.

### Stage 2: refinement of the STAR intervention content: meetings with health professionals

Questionnaire responses from stage 1 were summarised and discussed with 18 healthcare professionals in four meetings. Participants came from four UK orthopaedic centres and included orthopaedic surgeons (*n* = 10), physiotherapists (*n* = 4), nurses (*n* = 3) and one clinical co-ordinator. Nine of the participants were recruited as they completed consensus questionnaires in stage 1, and an additional nine participants were recruited through informal networks at the four centres. Meetings were facilitated by the study’s Chief Investigator (RGH) and sought to identify changes that could be made to the care pathway in light of clinicians’ experience of providing patient care within different organisational settings. These meetings, in conjunction with the questionnaire data, informed a number of substantive changes to the design of the care pathway (summarised in Table 2).

**Table 2:** Stage 1 and 2 – refinements made to the care pathway

Issue identified	Changes made to address this issue
Patients need to be identified earlier to ensure that treatment is initiated at 3 months post-operation.	Screening of patients to identify those with pain brought forward to 2 months post-operation to allow patients to be seen promptly at 3 months post-operation. A second screening process will occur prior to the assessment appointment to ensure that patients still have pain.
Patients who are offered nurse-led or self-monitoring need to be regularly followed up and referred to other services if needed.	‘Monitoring ‘changed to ‘follow-up’. Patients will be offered regular telephone follow-up with a health professional and further referrals if pain does not improve.
Physiotherapy should be a treatment option.	Physiotherapy included as a referral pathway.
There are additional ‘red flags’ that should initiate an urgent referral to a surgeon.	Knee stiffness or patellofemoral joint problems will initiate an urgent referral to an orthopaedic surgeon.
Patients with anxiety should be referred to their General Practitioner.	Signs of anxiety will initiate a General Practitioner referral for review and treatment.
Treatment of neuropathic pain should begin as soon as possible after assessment, ideally while waiting for pain clinic appointment.	Patients with signs of neuropathic pain will be referred to their General Practitioner to initiate medication treatment. If there is no improvement in 6 weeks, patients will be referred to a pain specialist.
Referrals to pain services needs to be via General Practitioner.	General Practitioners will be asked to request an urgent referral to pain services for patients who meet the diagnostic criteria for complex regional pain syndrome.

### Stage 3: testing intervention delivery and acceptability to patients

This phase involved evaluation of intervention delivery and acceptability to patients with chronic pain after total knee replacement. We sought to identify a small sample of patients with chronic pain after knee replacement to invite to attend an assessment clinic.

A screening questionnaire was sent to 196 patients who had a primary total knee replacement between 3 and 6 months previously at one elective orthopaedic centre. To identify individuals with potential chronic pain, patients with a score of 0–14 on the seven items of the Oxford Knee Score pain subscale [20, 21] were considered eligible for invitation to the STAR assessment clinic. This threshold was selected based on previous work using cluster analysis to identify people with moderate-severe pain after total knee replacement [22]. Completed screening questionnaires and consent forms were received from 72 (37%) patients. Of these, 15 patients (21% of responders) reported pain in their replaced knee *and* consented to further contact. All 15 were invited to attend an assessment clinic appointment on two occasions and to see two professionals, separately, at each clinic; ten agreed and attended.

At the first assessment clinic appointment, participants were assessed separately by a consultant orthopaedic knee surgeon with a specialist interest in post-operative knee pain and an extended scope practitioners (ESP: a registered allied healthcare professional with specialist training in orthopaedics). Each appointment lasted approximately 1–2 hours and the same processes were followed by each health professional. Details of the clinic assessment appointment are provided in Additional file 2. In brief, patients were asked to complete a questionnaire, have a detailed radiographic assessment of their replaced knee, and provide a blood sample to test for markers of infection. After taking a medical history, the health professional conducted a physical examination including an assessment of knee malalignment, tenderness, infection, stiffness, instability, and complex regional pain syndrome. Patients were then invited to attend a repeat assessment clinic 1–4 weeks later. In the second clinic, patients were reassessed by the same orthopaedic surgeon and ESP and the same processes were followed. The outcome of each assessment clinic appointment was recorded on a standardised proforma. After attending the second clinic appointment, patients were asked to complete a questionnaire about their experiences of the clinics to assess the acceptability of the assessment clinic process.

#### Changes to assessment procedures

This work highlighted the need for a number of changes to the design and processes of the assessment clinics. These involved the inclusion of an additional screening tool for neuropathic pain, provision of a goniometer (a device to measure angles) to assess range of motion and facilitate standardised assessment, further training on the assessment of complex regional pain syndrome, and guidance to ensure standardised radiographs were taken for the assessment of limb alignment. Further details of the issues identified and the steps taken to address these are provided in Table 3. This work also informed the content of a comprehensive intervention training manual and 1-day training event to be provided to all healthcare professionals intended to deliver the intervention that will be evaluated in a future randomised controlled trial. The intervention training manual consists of detailed standard operating procedures for the conduct and interpretation of assessments and guidance on appropriate referral pathways.

**Table 3:** Stage 3 – changes to the care pathway design and processes

Issue identified	Changes made to address this issue
DN-4 (neuropathic pain measure) did not identify all patients with clinical symptoms of a neuropathic component to their pain.	Addition of PainDETECT to the clinic assessment questionnaire and trial questionnaires.
Inconsistencies in the assessment of knee stiffness between the orthopaedic surgeon and extended scope practitioner.	Extended scope practitioners to be provided with a goniometer for the assessment of range of motion.
Complexity around diagnosing complex regional pain syndrome.	The intervention training manual and the training session will highlight the importance of referring patients if complex regional pain syndrome is suspected, even if formal criteria are not met, to allow for a specialist assessment.
X-ray film was inadequate to allow assessment of alignment (due to rotation).	Guidance provided in the intervention training manual on standardised wording to use when ordering X-rays. Training will be provided to extended scope practitioners on how to identify a rotated X-ray film.

#### Acceptability of assessment clinics

Eight patients completed the questionnaire about the acceptability of the assessment clinics. Participants thought that the content of the clinic was acceptable and appropriate (Table 4). Four patients provided free-text comments indicating that they found clinics to be informative and interesting. A plain English summary of the findings, prepared with input from a patient and public involvement forum [14], was sent to participants.

**Table 4:** Stage 3 – patient responses (*n* = 8) to questions about the acceptability of the assessment clinics

	Yes	No	Comments
Did you receive adequate information about the clinics beforehand?	8	0	
Did you feel that it was appropriate that this appointment was held at a hospital?	7	1	Parking at hospital difficult
Was the length of the clinic acceptable?	6	2	Surgeon delayed because previous clinic overran
Were the examinations that were performed on your knee acceptable?	8	0	
Were the written questionnaires that you completed during the clinic acceptable?	7	1	Some questions seemed contradictory
Were the questions that you were asked by the health professional acceptable?	8	0	
Are there any aspects of the clinics that you think could you improved?	1	7	The amount of time spent waiting

### Stage 4: evaluating stakeholders’ views about implementation of the STAR care pathway

To assess views about implementation of the STAR trial intervention, 15 health professional stakeholders involved in the development or future delivery of the intervention were invited to complete an online questionnaire. The questionnaire was based on the Normalisation Measure Development (NoMAD) instrument for measuring implementation processes from the perspectives of stakeholders involved in the implementation work [23]. The 23-item NoMAD instrument has 20 core construct items that reflect the four core constructs of Normalisation Process Theory (NPT). These represent different kinds of work that people do when implementing a new practice: coherence (how people make sense of the new intervention), cognitive participation (how people build a community of practice around a new intervention), collective action (the operational work that people do to enact a new intervention), and reflexive monitoring (people’s appraisal of how a new intervention affects them) [24]. The instrument also includes three general questions about the intervention. Authors of NoMAD advise that the questions in the instrument template are adapted to ensure that they make sense to the participants in the context of the study, but that the adaptation is done in such a way that the overall meaning of the questions is not changed (e.g. incorporating the name of the intervention into the question). Ten stakeholders from across the UK with experience in management of chronic pain after knee replacement completed the survey. Stakeholders were consultant orthopaedic surgeons (*n* = 4), physiotherapy ESPs (*n* = 3), a medical psychologist, a consultant nurse and a pain psychology advisor. An electronic link to the survey was sent to each stakeholder along with a draft copy of the intervention training manual. Responses to each of the survey questions were then collated and a descriptive summary of the responses to each question was developed.

Stakeholders’ opinions varied about how different the STAR care pathway was to usual patient care. This may reflect diversity in current practice for assessment, management and treatment of chronic post-surgical pain [7]. Professionals with orthopaedic expertise were familiar with the tests used within the clinic, such as assessment of complex regional pain syndrome and post-operative wound infection, as well as examinations of knee tenderness, alignment, instability and range of motion. All stakeholders felt that the pathway components could become incorporated within usual patient care at their hospital. In terms of how stakeholders made sense of the STAR care pathway, there was a generally high level of coherence. Those working in an orthopaedic setting could see how the pathway differed from their usual ways of working. Stakeholders who had been involved in earlier stages of care pathway development agreed that there was a shared understanding among their clinical colleagues of its purpose. The ESPs who had not been involved in earlier stages but who would deliver the assessment clinics in the future trial neither agreed nor disagreed with this. Planned training prior to the start of the trial will help ESPs to feel secure in their delivery of the STAR trial intervention.

All stakeholders understood how the care pathway would affect the nature of their own work, and the specific tasks and responsibilities around the new practices. All stakeholders could identify key individuals in their places of work who would drive delivery of the new care pathway forward. Consultant orthopaedic surgeons and ESPs thought that contributing to and delivering the care pathway would be a legitimate part of their clinical role and they were open to working with colleagues in new ways and would continue to support the development of the care pathway. Some stakeholders (two surgeons and one ESP) indicated that problems might arise when trying to integrate the new practices into their existing work, which as noted previously may reflect variation in current usual care. However, stakeholders were confident that the new practices would not disrupt their current working relationships and were generally confident that the right people with the right skills would be delivering the STAR care pathway. Stakeholders also thought that sufficient training and resources would be available to support the delivery of the STAR care pathway, and most thought that it would be adequately supported by hospital management.

All stakeholders were aware of the proposed benefits of the intervention for patients, and most thought that their colleagues felt the STAR care pathway was worthwhile. However, some were less certain about this, which indicates that further work may be needed to promote the new practices among colleagues. Stakeholders generally thought that the intervention would have a valuable effect on their work. Most indicated that feedback about the STAR care pathway could be used to improve it in the future and all stakeholders could see how they could modify how they worked with the intervention.

### The STAR trial intervention

Based on the four stages of work described above, the STAR trial intervention was refined and prepared for evaluation within the context of a multicentre randomised controlled trial. Here we report the finalised intervention in keeping with guidance from TIDieR [16]; the TIDieR checklist is provided in Additional file 3. A schematic depiction of the final STAR intervention is provided in Additional file 4 and further details of the intervention are provided in Additional files 4 and 5.

In summary, the STAR care pathway intervention consists of an assessment clinic for patients with pain at 3 months after total knee replacement and then onward referral to existing, appropriate services, with up to six follow-up telephone calls over 12 months. In the assessment clinic, participants are assessed by a trained ESP. The ESP will be experienced in the general assessment of knee replacement patients and additionally will have attended a 1-day training event on the care pathway and have been provided with a comprehensive intervention training manual. The face-to-face assessment involves the ESP taking a clinical history, reviewing patient-completed questionnaires, conducting a knee examination, reviewing radiographs and blood test results. Patient-completed questionnaires include the Brief Pain Inventory [25], Hospital Anxiety and Depression Scale [26], PainDETECT [27] and Douleur Neuropathique 4 [28]. The knee examination involves evaluating sites and nature of knee tenderness, surgical wound healing, range of motion, alignment, stability, patellofemoral joint function, signs of infection and signs and symptoms of complex regional pain syndrome as per the Budapest criteria [29]. A blood sample is taken to test for markers of infection. Patients have anteroposterior long leg alignment, lateral and patella skyline knee radiographs taken if these have not already been performed as part of their usual care. This is to evaluate alignment and assess for evidence of fracture or concerns with sizing, fixation or position of the implants. The appointment lasts up to 1 hour, although additional time may be required for radiographs.

On the basis of the findings of assessment, patients are referred to appropriate existing care for further treatment, which may include one or more of the following: an orthopaedic surgeon; a physiotherapist; a GP for treatment of depression or anxiety; and/or pain specialists for neuropathic pain or complex regional pain syndrome (via GPs). Follow-up is also available if this is appropriate. Patients will also receive telephone follow-up from the ESP, up to a maximum of six times over 12 months, and further referrals can be made as required. Copies of all referral letters are sent to the patient, their treating orthopaedic surgeon and their GP. The STAR care pathway is individualised and flexible, and other referrals can be made depending on the needs of the patient.

## Discussion

This article describes a comprehensive programme of research undertaken to refine the design of a complex intervention before rigorous evaluation within the framework of a multicentre randomised controlled trial. The MRC recommends that key elements of the development and evaluation process for complex interventions are development, feasibility and piloting, evaluation and implementation [30], with the MRC’s more recent guidance placing greater emphasis on the early-phase development and piloting work [15]. Although there is comprehensive guidance to inform conduct of the latter three elements of this process, there is little guidance about appropriate methods for intervention development [31]. Robust intervention development is needed to minimise weaknesses in design and subsequent difficulties in evaluation and implementation [15]. This research adds to the literature by providing a detailed account of work undertaken to develop and refine a complex intervention in an orthopaedic setting. The intervention development work focused on refinement of content and allowed logistical issues with intervention delivery to be identified and addressed. This research also informed the design of an intervention training package and evaluated views about implementation of the intervention from the perspectives of stakeholders and those who will deliver the intervention at each site during the trial.

Undertaking intervention development work is time consuming and labour intensive [32]. However, this study highlights the importance of conducting such preparatory work to optimise the design and delivery of a complex intervention prior to evaluation within a randomised trial. Engagement with clinicians through consensus work in stage 1 ensured that the components of the intervention were appropriate and identified modifications to further improve the intervention design. Discussions with experienced clinicians from the future trial centres in stage 2 provided an opportunity for people involved in the delivery of the trial to shape the intervention, helping to foster and strengthen their engagement in the research [31]. Testing intervention delivery in stage 3 provided the opportunity to identify any problems with delivering the intervention and to formulate and implement solutions to these, thereby minimising the need for logistical modifications during the pilot phase of the trial. This also informed the development of training materials to ensure that staff will be appropriately equipped to deliver the intervention within the trial. Delivering the intervention allowed the acceptability of the clinic process to be evaluated with patients. This is particularly important in trials of complex interventions that can face challenges to their delivery because of low intervention uptake and attendance [33, 34]. The final stage of this research (stage 4) involved evaluating views about implementation of the intervention, which demonstrated that stakeholders understood the intervention and were aware of the proposed benefits of the intervention for patients. They could see how the intervention would affect the nature of their own work and were confident that they could deliver the intervention and that it could become normalised within their own practice; they also thought that it would have a valuable effect on their work. This highlighted that training provided to ESPs should include focusing on helping them to feel more confident in delivering the intervention.

Approaches to intervention development work need to be flexible and adapted to the clinical context and setting. Although the work to develop the STAR care pathway was conducted in a manner that was designed to be comprehensive and context appropriate, we acknowledge that there was potential for additional work that we did not undertake. For instance, additional methods might have included economic modelling [35, 36], direct observation of intervention delivery [37] and factorial screening experiments [38]. Follow-up telephone interviews with stakeholders who completed the NoMAD questionnaire in stage 3 may have provided further insight and elaboration around views on implementation of the intervention. In the design of this multistage study, we chose to focus on ensuring that the intervention was deliverable, implementable and acceptable. This was achieved within a relatively tight 12-month window to ensure that the future trial could be conducted within a reasonable timeframe to enable impact on future patient care. Therefore, the methods we chose were designed to focus on answering these key questions. Also, assessment of the delivery of the STAR care pathway in stage 3 was conducted in only one centre. Although we anticipate that lessons learnt in the single centre will be transferable to other centres, research at multiple centres may have identified additional logistical issues. Despite these limitations, we have confidence that the STAR complex intervention has been refined in keeping with the spirit of MRC guidance, and the future trial will include an internal pilot phase that will enable further small refinements to the intervention if needed.

## Conclusion

Conducting feasibility or pilot studies before definitive trials to evaluate complex interventions can inform and improve the design and delivery of randomised trials [39]. Feasibility and pilot studies address trial design issues, such as recruitment, retention, outcome assessment, adherence and uptake to intervention and sample size calculation. Of equal importance to trial design is intervention development. With increasing awareness about the need to design and develop interventions in a robust fashion to maximise appropriateness and smooth implementation, there is a need for more guidance about how best to undertake intervention development in a manner that is rigorous, appropriate and efficient. Our study provides an example of the methods that can be used to address key questions within intervention design in a relatively tight timeframe. The next stage of this programme of research is to evaluate the clinical and cost-effectiveness of the STAR care pathway in a definitive multicentre randomised trial, which will include an internal pilot phase (ISRCTN92545361). Details of the design of this randomised trial will be published separately as a trial protocol paper.

## Additional files

Additional file 1: Schematic depiction of the draft STAR trial intervention. (DOCX 39 kb)

Additional file 2: Overview of STAR assessment clinic appointment, telephone follow-up and recommended treatment referral pathways. (DOCX 18 kb)

Additional file 3: The Template for intervention description and replication (TIDieR) checklist. (DOCX 15 kb)

Additional file 4: Schematic depiction of the final STAR intervention. (DOCX 50 kb)

Additional file 5: Summary of the STAR trial intervention using TiDIER criteria. (DOCX 15 kb)

## Abbreviations

ESP: Extended scope practitioner
GP: General practitioner
MRC: Medical Research Council
NHS: National Health Service
NoMAD: Normalisaton Measure Development
NPT: Normalisation Process Theory
STAR: Support and Treatment After joint Replacement
TIDieR: Template for intervention description and replication
