# Supplementary material for: Development of a complex intervention for people with chronic pain after knee replacement: the STAR care pathway
Source: Trials. 2018 Jan 23;19:61. doi: 10.1186/s13063-017-2391-8 (PMC5781277; doi:10.1186/s13063-017-2391-8)
Supplement: Supplementary file 1 — Schematic depiction of the draft STAR trial intervention. (DOCX 39 kb) [file 13063_2017_2391_MOESM1_ESM.docx]

Patients with moderate or severe pain at 3 months after total knee replacement (identified through either the Brief Pain Inventory or WOMAC pain scale)

Pain assessment and care allocation (nurse or extended scope practitioner led)

Feedback and re-referral

Monitoring (nurse and self)

Treatment or referral

Treatment that might include neuropathic pain pathway or CRPS pathway as appropriate

Pain improves

Pain persists or is problematic (self-completed questions)

Surgeon

Pain specialist

GP

Major depression

Severe or interfering pain with indications of neuropathic or CRPS elements

Surgery

Signs of infection, misalignment or instability

No further treatment

Red flags

**Additional file 1: Schematic depiction of the draft STAR trial intervention**
