# Supplementary material for: Development of a complex intervention for people with chronic pain after knee replacement: the STAR care pathway
Source: Trials. 2018 Jan 23;19:61. doi: 10.1186/s13063-017-2391-8 (PMC5781277; doi:10.1186/s13063-017-2391-8)
Supplement: Supplementary file 2 — Overview of STAR assessment clinic appointment, telephone follow-up and recommended treatment referral pathways. (DOCX 18 kb) [file 13063_2017_2391_MOESM2_ESM.docx]

**Additional file 2: Overview of STAR assessment clinic appointment, telephone follow-up and recommended treatment referral pathways**

| **Component** | **Overview of procedure** | **Recommended referrals** |
| --- | --- | --- |
| **History and symptoms** | Patients are asked about the details of their pre-operative pain, knee replacement surgery, any post-operative complications, their recovery so far, expectations and satisfaction with surgery, general health, co-morbidities, other painful sites/conditions, analgesia used, and social circumstances. The onset, duration, nature, location and impact of the pain should be explored. | To be used in conjunction with results of other assessments to inform referral decisions. |
| **Review of validated patient-reported outcome measures** | The patient is asked to complete the Brief Pain Inventory, PainDETECT, Douleur Neuropathique 4 and Hospital Anxiety and Depression Score immediately prior to seeing the Extended Scope Practitioner. These are reviewed during the assessment clinic. | ***Brief Pain Inventory***  To be used in conjunction with results of other assessments to inform referral decisions.  ***PainDETECT and DN4***  Patients with possible or probable on either questionnaire neuropathic pain component should be referred to GP to initiate neuropathic pain medications. If patients are already on neuropathic pain medications, then liaise with GP to: trial an alternative, increase the dose of pain medication, or refer to pain clinic for additional assessment and management.  ***Hospital Anxiety and Depression Score***  Patients with probable or definite depression or anxiety should be referred to their GP. If already taking medication, liaise with GP to consider modification or alteration to treatment. |
| **Knee palpation tenderness** | Patients are asked about areas of knee tenderness. Light palpation on all knee areas is performed to evaluate generalised hypersensitivity or hyperaesthesia. All knee areas are lightly rubbed to identify any sites of allodynia. The knee is palpated in a systematic manner for tenderness. | To be used in conjunction with results of other assessments to inform referral decisions.  -Global tenderness may suggest a non-surgical origin for pain.  -Focal tenderness may suggest a surgical origin for pain.  -Hyperaesthesia or allodynia may suggest a neuropathic pain component or Complex  Regional Syndrome |
| **Wound assessment** | Assessment of healing, including ooze, redness, residual scab, retained stitch, dehiscence, ulceration, inflammation and hypertrophy is conducted. Skin temperature is evaluated using the backs of hands on both knees. Patients are asked about symptoms on fever, rigors, sweats, change in appetite, fatigue and generalised malaise. A blood sample is taken and sent to lab for testing of C-Reactive Protein levels. | Clinical suspicion for infection PLUS an elevated CRP level requires urgent surgeon referral.  Minimal clinical suspicion for infection but raised CRP requires repeat of blood test approximately one month later. |
| **Range of motion** | Patient’s ability to perform a straight leg raise is assessed to give a guide to extensor mechanism and quadriceps function. Assessment of passive range of movement is made with a goniometer. | Extension deficit >10^o^, excessive hyperextension > 10^o^ or flexion < 85^o^ is sufficient for surgeon referral.  Lack of active extension or a gross extensor lag may suggest a significant extensor mechanism problem, which would warrant a surgeon referral. |
| **Stability** | Knee stability is assessed with the patient lying supine on a couch with a single pillow under their head. If any test is positive, it is graded as 1+/2+/3+ (5mm/10mm/15mm).  A posterior draw test is performed. The knee is flexed as close to 90^o^ as the patient can tolerate. Both hands are placed around the proximal tibia and a posterior force is applied to the proximal tibia.  Varus/valgus stability is assessed by supporting the knee and holding it flexed at 30^o^. To assess varus stability, one hand is placed on the medial aspect of the knee with the other around the lateral aspect of the ankle, and the knee stressed in a varus direction to assess the degree of opening. Valgus stability is assessed in the same way but with a hand on the lateral knee and medial ankle. | 2+ or greater instability in any direction is sufficient for surgeon referral. |
| **Patellofemoral joint** | A patellofemoral compression test is performed. Thumbs are applied to the anterior patella and it is gently compressed towards the trochlea groove. Whilst pressure is being applied, the patient is asked to carefully contract their quadriceps muscles. A positive test is discomfort elicited on quadriceps contraction.  Patellofemoral tracking is assessed through range of flexion and extension and peripatellar tenderness on palpation should give an indication of potential patellofemoral joint concerns. | Any concern for patellar maltracking or subluxation should prompt surgeon referral.  If the patellofemoral compression test is strongly positive, consider referral to surgeon for assessment of patellofemoral problems |
| **Complex Regional Pain Syndrome (CRPS)** | History and examination findings prior to this stage of assessment will inform the possibility of a diagnosis of CRPS. Patients are assessed for pain that is spreading/radiating away from the joint, allodynia or touch sensitivity of the skin, swelling of the limb, colour changes or abnormal hair growth. | If any of the above signs or symptoms are present, this should be sufficient to raise suspicion of a potential CRPS diagnosis and formal criteria should be assessed. Extended Scope Practitioners are provided with the formal CRPS criteria.  If CRPS is suspected but the formal criteria are not met, patients should be referred to their GP to begin neuropathic pain medications, and recommend referral onto pain clinic if minimal or no response to analgesia. If formal CRPS criteria are met, patients should be referred to the pain services via their GP. Physiotherapy should be continued in either of the above scenarios, so as to encourage normal use and touch of the limb, and improvement in function despite the limitations of pain. |
| **Radiographs** | Patients are x-rayed before attending the clinic appointment. For radiographic assessment a patient requires an AP weight-bearing long-leg alignment film, a true lateral and a patella skyline. It is important to ensure that the radiograph is not rotated.    Coronal alignment is assessed by measuring the hip-knee angle on the long-leg film. This involves measurement of the angle from the centre of the femoral head to the distal centre of the femoral knee prosthesis and on from here to the centre of the ankle.  The radiographs should also be evaluated for any evidence of fracture, or concerns with sizing, fixation or position of the implants. | If there is any gross concern regarding alignment, fracture, sizing, fixation or position of implants, referral to a surgeon should be considered. |
| **Additional information relevant to the clinic assessment appointments** | Further details of all assessments are provided in the intervention training manual and all results should be recorded on the standardised assessment proforma. | All referrals should be considered in the wider context and referrals additional to those outlined in the intervention training manual can be performed depending on the needs of the individual patient. |
| **Telephone follow-up** | All patients who attend a clinic appointment have a maximum of six follow-up telephone consultations. This is to follow-up on the care that patients are receiving and to ensure that any referrals are being undertaken. Additionally, further referrals can be made on the basis of these telephone follow-up consultations. Details of these telephone calls are recorded on a standardised proforma. | Not all patients with pain will meet criteria for, or indeed will need, onward referral. A proportion of patients will have pain that will simply improve with time and it is entirely appropriate that such patients are followed up by telephone to monitor progress. If necessary, referrals can be made at a later date after telephone follow-up consultations. All patients who are being monitored without referral should have a telephone follow-up consultation at six weeks, three months and six months after their clinic appointment. |
