# Supplementary material for: Development of a complex intervention for people with chronic pain after knee replacement: the STAR care pathway
Source: Trials. 2018 Jan 23;19:61. doi: 10.1186/s13063-017-2391-8 (PMC5781277; doi:10.1186/s13063-017-2391-8)
Supplement: Supplementary file 3 — The Template for intervention description and replication (TIDieR) checklist. (DOCX 15 kb) [file 13063_2017_2391_MOESM3_ESM.docx]

**Additional file 3: The TIDieR (Template for Intervention Description and Replication) Checklist**

| **Item number** | **Item** | **Where located** |
| --- | --- | --- |
|  | **BRIEF NAME** |  |
| **1.** | Provide the name or a phrase that describes the intervention. | Title page |
|  | **WHY** |  |
| **2.** | Describe any rationale, theory, or goal of the elements essential to the intervention. | Introduction |
|  | **WHAT** |  |
| **3.** | Materials: Describe any physical or informational materials used in the intervention, including those provided to participants or used in intervention delivery or in training of intervention providers. Provide information on where the materials can be accessed (e.g. online appendix, URL). | Additional file 4 |
| **4.** | Procedures: Describe each of the procedures, activities, and/or processes used in the intervention, including any enabling or support activities. | Additional file 5 |
|  | **WHO PROVIDED** |  |
| **5.** | For each category of intervention provider (e.g. psychologist, nursing assistant), describe their expertise, background and any specific training given. | Additional file 4 |
|  | **HOW** |  |
| **6.** | Describe the modes of delivery (e.g. face-to-face or by some other mechanism, such as internet or telephone) of the intervention and whether it was provided individually or in a group. | Additional file 4 |
|  | **WHERE** |  |
| **7.** | Describe the type(s) of location(s) where the intervention occurred, including any necessary infrastructure or relevant features. | Additional file 4 |
|  | **WHEN and HOW MUCH** |  |
| **8.** | Describe the number of times the intervention was delivered and over what period of time including the number of sessions, their schedule, and their duration, intensity or dose. | Additional file 4 |
|  | **TAILORING** |  |
| **9.** | If the intervention was planned to be personalised, titrated or adapted, then describe what, why, when, and how. | Additional file 4 |
|  | **HOW WELL** |  |
| **11.** | Planned: If intervention adherence or fidelity was assessed, describe how and by whom, and if any strategies were used to maintain or improve fidelity, describe them. | Additional file 4 |
