# Supplementary material for: Development of a complex intervention for people with chronic pain after knee replacement: the STAR care pathway
Source: Trials. 2018 Jan 23;19:61. doi: 10.1186/s13063-017-2391-8 (PMC5781277; doi:10.1186/s13063-017-2391-8)
Supplement: Supplementary file 4 — Schematic depiction of the final STAR intervention. (DOCX 50 kb) [file 13063_2017_2391_MOESM4_ESM.docx]

Additional file 4: Schematic depiction of the final STAR intervention

Pain improves

No further treatment

Treatment or referral

Surgeon with urgent referral

GP

Assessment +/- Surgery

Follow-up

Physiotherapy

Depression or anxiety

Severe or interfering pain with indications of Neuropathic Pain

Signs of infection, malalignment, stiffness, PFJ issue or instability

Severe or interfering pain with indications of CRPS

Treatment that might include neuropathic pain pathway as appropriate

Pain specialist

GP to initiate medication

No Improvement

Pain re-assessment after 6/52

Patients with moderate or severe pain at 2 months after total knee replacement (identified through the Oxford Knee Score pain scale)

Pain assessment and care allocation by extended scope practitioner at 3 months after total knee replacement

Follow up and re-referral (all patients to be telephoned up to 6 times over 12 months)

Pain specialist

Urgent if meets CRPS diagnostic criteria

Treatment that might include CRPS pathway as appropriate

GP to initiate urgent referral
