# Supplementary material for: Development of a complex intervention for people with chronic pain after knee replacement: the STAR care pathway
Source: Trials. 2018 Jan 23;19:61. doi: 10.1186/s13063-017-2391-8 (PMC5781277; doi:10.1186/s13063-017-2391-8)
Supplement: Supplementary file 5 — Summary of the STAR trial intervention using TiDIER criteria. (DOCX 15 kb) [file 13063_2017_2391_MOESM5_ESM.docx]

**Additional file 5: Summary of the STAR trial intervention using TiDIER criteria**

| **TiDIER Criteria** | **Description of STAR trial intervention** |
| --- | --- |
| **Name** | STAR (Support and Treatment After joint Replacement): A care pathway for patients with chronic pain after knee replacement |
| **Rationale** | Providing a clear entry point to services and delivering combination treatments that are matched to individual patients’ pain characteristics may lead to improved outcomes for patients with chronic pain after knee replacement. |
| ***Participant referral to intervention*** | |
| **Participants receiving STAR intervention** | Patients who are three months after primary total knee replacement for osteoarthritis and have pain in their operated knee, defined as a score of ≤14 on the 7 pain items of the Oxford Knee Score. |
| **Referral procedure** | Participant invited to attend a 1-hour individual outpatient hospital assessment clinic appointment with a physiotherapy Extended Scope Practitioner. Written letter to confirm appointment location, time and date. |
| ***Staff training for intervention delivery*** | |
| **Training format** | Three hour interactive training session and comprehensive intervention training manual. |
| **Who provided training** | Consultant Orthopaedic Surgeon with specialist expertise in knee surgery and post-graduate qualification in chronic pain after knee replacement. |
| **Who received training** | Physiotherapy Extended Scope Practitioners delivering the intervention. |
| ***Assessment Procedure*** | |
| **Materials required** | Assessment clinic appointment: Goniometer, bed or plinth and pillow, venipuncture equipment, intervention training manual, patient-completed questionnaires, standardised assessment proforma, formal CRPS criteria.  Telephone follow-up: telephone, intervention training manual, standardised proforma. |
| **Where** | Assessment clinic appointment undertaken in a quiet hospital room. Room must have bed or plinth to allow patient to lie in supine position. Proximity to radiology department should be considered. |
| **When** | Single 1-hour assessment clinic appointment when participant is three months post-operative. Telephone follow-up up to six times over 12 months to be arranged as deemed appropriate by the Extended Scope Practitioner. |
| **Tailoring** | Every assessment conducted on every participant. Further assessment and onward referral arranged as appropriate (details in Additional file 4). Referral pathways can be tailored to individual patients and multiple referrals can be undertaken. Further referrals can be initiated as needed after follow-up telephone calls. Referrals not listed in the intervention training manual can be made depending on the needs of the patient. |
| **Modifications** | Intervention refined during intervention development work (see Additional files 1 and 2) |
| ***Intervention Fidelity*** | |
| **Training** | Training signature logs completed after attendance at training session. |
| **Intervention delivery** | Training emphasises adherence to the intervention training manual. During the randomised controlled trial, Quality Control (QC) visits to staff delivering the intervention at every site will be undertaken by a member of STAR team. QC visits will include observation of the delivery of the assessment clinic appointment and follow-up telephone calls. A minimum of one STAR assessment per Extended Scope Practitioner will be observed. The observer will complete QC assessment form to record accuracy of completion of trial paperwork; checklist of assessment of risk factors; whether referrals were warranted and actioned appropriately. Any areas requiring further training will be highlighted and actioned. QC form will be signed and dated by assessor and Extended Scope Practitioner. |
